# First Identification of Human Adenovirus Subtype 21a in China With MinION and Illumina Sequencers

**DOI:** 10.3389/fgene.2020.00285

**Published:** 2020-04-07

**Authors:** Fuqiang Ye, Yifang Han, Juanjuan Zhu, Peng Li, Qi Zhang, Yanfeng Lin, Taiwu Wang, Heng Lv, Changjun Wang, Chunhui Wang, Jinhai Zhang

**Affiliations:** ^1^Department of Disease Control and Prevention, Center for Disease Control and Prevention of Eastern Theater Command, Nanjing, China; ^2^School of Life Science and Technology, China Pharmaceutical University, Nanjing, China; ^3^Center for Infectious Disease Control, Center for Disease Control and Prevention of People’s Liberation Army of China, Beijing, China

**Keywords:** human adenovirus subtype 21a, adenovirus infection, pathogen detection, MinION sequencing, Illumina sequencing

## Abstract

Human adenoviruses (HAdVs) have been demonstrated to cause a diversity of diseases among children and adults. The circulation of human adenovirus type 21 (HAdV21) has been mainly documented within closed environments in several countries. Nonetheless, respiratory infections or outbreaks due to HAdV21 have never been reported in China. MinION and Illumina platforms were employed to identify the potential pathogen from a throat swab. Discrepancies between MinION and Illumina sequencing were validated and corrected via polymerase chain reaction (PCR). Genomic characterization and recombinant event detection were then performed. Among the 35,466 high-quality MinION reads, a total of 5,999 reads (16.91%) could be aligned to HAdV21 reference genomes (genome sizes ≈35.3 kb), among which 20 had a length of >30 kb. A genome sequence assembled from MinION reads was further classified as HAdV subtype 21a. Random downsampling revealed as few as 500 nanopore reads could cover ≥96.49% of current genome. Illumina sequencing displayed good consistency (pairwise nucleotide identity = 99.91%) with MinION sequencing but with 31 discrepancies that were further validated and confirmed by PCR coupled with Sanger sequencing. Restriction enzymes such as *Bam*HI and *Kpn*I were able to distinguish the present genome from HAdV21 prototype and HAdV21b. Phylogenetic analysis employing whole-genome sequences placed our genome with members only from subtype 21a. Common features among HAdV21a strains were identified, including polymorphisms discovered in penton and 100 kDa hexon assembly–associated proteins and a recombinant event in the E4 gene. Using MinION and Illumina sequencers, we identified the first HAdV21a strain from China, which could provide key genomic data for disease control and epidemiological investigations.

## Introduction

Since the first discovery by Rowe et al. (1953), human adenoviruses (HAdVs) have been found to cause a diversity of diseases in children and adults (Radke and Cook, 2018; Prusinkiewicz and Mymryk, 2019; Yao et al., 2019). Belonging to the Mastadenovirus genus in the Adenoviridae family, HAdVs are double-stranded DNA viruses with 35–36 kb pairs in their genomes (Radke and Cook, 2018). At present, almost 90 Adv subtypes distributed across seven species (termed as A through G) have been classified, among which 55 could lead to human infections (Prusinkiewicz and Mymryk, 2019; Yao et al., 2019). A variety of tissue tropisms are observed in these HAdVs, with preferred infections of species B/C/E in the respiratory tracts, species B/C/D/E in the conjunctiva, species A/F/G in the gastrointestinal tract, and species B in the kidney (Lenaerts et al., 2008; Lion, 2014; Prusinkiewicz and Mymryk, 2019). Human adenovirus types 3, 4, 7, 11, 14, 21, and 55 are common AdV pathogens responsible for human respiratory tract infections or respiratory outbreaks in many regions (Lewis et al., 2009; Kajon et al., 2010; Li et al., 2014; Scott et al., 2016; Bautista-Gogel et al., 2019; Prusinkiewicz and Mymryk, 2019; Sammons et al., 2019; Zhang et al., 2019). Clinical symptoms caused by HAdVs are usually mild and self-limiting in healthy people; however, immunologically compromised individuals such as young children, elderly persons, and immunosuppressed patients might have a higher risk of severe disease and even death (Ison, 2006; Kandel et al., 2010; Lee et al., 2010; Scott et al., 2016; Pfortmueller et al., 2019). Notably, HAdV-associated respiratory outbreaks are commonly seen in closed population clusters, including schools and hospitals (Scott et al., 2016; Radke and Cook, 2018).

The circulation of human adenovirus type 21 (HAdV21) has been mainly documented in care facilities, military hospitals, or military recruit training centers (Hage et al., 2014; Kajon et al., 2015; Scott et al., 2016; Philo et al., 2018; Pfortmueller et al., 2019). Human adenovirus type 21 can cause upper respiratory tract infection, pneumonia, hemorrhagic cystitis, myocarditis, and encephalitis. There are currently three HAdV21 subtypes according to the restriction enzyme analysis (REA), HAdV subtype 21a (HAdV21a), HAdV21b, and HAdV21p (Kajon et al., 2015). As noted above, HAdV21 commonly leads to mild clinical symptoms such as fever, cough, shortness of breath, fatigue, nausea, rhinorrhea, and myalgia (Scott et al., 2016). However, HAdV21a is linked to severe pneumonia or acute respiratory distress syndrome, with some fatal outcomes (Hage et al., 2014; Pfortmueller et al., 2019).

Nanopore sequencing technology was made public by Oxford Nanopore Technologies (ONT) in 2014. When DNA or RNA module translocates through a nanopore, an ionic current will be produced from a constant voltage bias, and then a change in the ionic current can be observed (van Dijk et al., 2018). Nucleic acid bases can then be basecalled by built-in or the third-party basecallers. When compared to the next-generation sequencing (NGS) methods, nanopore sequencing technology has advantages such as real time, long reads, short turnaround time, and simple sample preparation procedures (van Dijk et al., 2018; Ameur et al., 2019). In particular, the ONT platform MinION, also characterized by portability and low cost in addition to the above advantages, can well handle rapid pathogen detection in the field (McIntyre et al., 2016; Castro-Wallace et al., 2017; Johnson et al., 2017; Edwards et al., 2019). MinION has been widely employed in pathogen identification from human clinical samples to identify Chikungunya virus, hepatitis C virus, and enterovirus (Wang et al., 2017; Imai et al., 2018; Xu et al., 2018). It has also played a vital role in rapid pathogen confirmation in recent epidemic outbreaks caused by Ebola virus (Quick et al., 2016), Zika virus (Faria et al., 2017; Quick et al., 2017), yellow fever virus (Faria et al., 2018), and Lassa virus (Kafetzopoulou et al., 2019), highlighting the value of MinION in microbial investigation and disease control.

Human adenovirus type 21 infections or respiratory outbreaks have been reported in Germany (Hage et al., 2014), Denmark (Barnadas et al., 2018), Switzerland (Pfortmueller et al., 2019), Argentina (Barrero et al., 2012), and the United States (Kajon et al., 2015; Scott et al., 2016). However, respiratory infections due to HAdV21 or related strains have rarely been reported in China to date. In this study, MinION and Illumina sequencers were used to obtain HAdV21 genome sequences from viral cultures of a swab sample from a patient with respiratory tract infection. Polymerase chain reaction (PCR) products were then used to validate and correct discrepancies between MinION and Illumina sequencing. The genomic characterization and phylogenetic analysis were further performed with the corrected HAdV21a genome.

## Materials and Methods

### Sample Collection, Initial Pathogen Screening, and Viral Cultivation

A throat swab sample was collected from a 20-year-old male outpatient with fever and sore throat in March 2019. The swab sample was immediately placed into preservation solution (Yocon, Beijing, China) and stored at -80°C until further processing. Initial real-time PCR tests of viral respiratory pathogens including HAdV, coronavirus, and influenza A/B viruses obtained a positive result for HAdV by using universal primers. However, PCR primers for epidemic HAdV subtypes in China, including HAdV1, HAdV3, HAdV4, HAdV7, HAdV14, and HAdV55, displayed negative results. The sample was cultivated in HEp-2 cell lines (ATCC, Manassas, VA, United States) supplemented with Dulbecco modified eagle medium (Life Technologies, Carlsbad, CA, United States) containing 2% fetal bovine serum (Life Technologies) until 90% cytopathic effects were visible. The patient provided written informed consent upon enrollment. The study conformed to the ethical guidelines of the 1975 Declaration of Helsinki and was approved by the Institutional Review Board of the Center for Disease Control and Prevention of Eastern Theater Command.

### DNA Extraction and Purification

Viral DNA was extracted from the cell culture supernatant by using a QIAamp^®^ MinElute^®^ Virus Spin Kit (QIAGEN, Germantown, MD, United States) following the manufacturer’s protocols. Viral DNA was subsequently cleaned up and concentrated with AMPureTM XP beads (Beckman Coulter, Indianapolis, IN, United States). The purified DNA was further tested by quantitative PCR for detection of the target virus.

### MinION Library Construction and Sequencing

A total of 452 ng viral DNA was used for MinION sequencing. The MinION library was constructed with the Rapid Sequencing Kit (SQK-RAD004) according to the manufacturer’s instructions. The library was loaded to a SpotON Flow Cell (FLO-MIN106). Then the control software MinKNOW (v3.1.19, downloaded from the ONT website with a custom account) was set up with a run time of 48 h. The basecalling of fast5 files to fastq files during sequencing was also turned on.

### Illumina Library Construction and Sequencing

Total DNA was sheared into ∼350 bp fragments by the Covaris ultrasonicator (Covaris, Woburn, MA, United States). A-tailed, ligated to paired-end adaptors and PCR amplified with a 350 bp insert were used for the library construction. Qubit 2.0 (Life Technologies) was then used to quantify the concentration of the library, and the insert size was tested with Agilent Bioanalyzer 2100 (Agilent, Santa Clara, CA, United States). Illumina NovaSeq 6000 platform (Illumina, San Diego, CA, United States) was used to generate 2 × 150 bp pair-end reads.

### PCR Validation and Correction

To validate and correct the genomic discrepancies between MinION and Illumina sequencing, we designed seven pairs of PCR primers covering these discrepant sites via Primer Premier 5.0 software. The PCR reaction was carried out in a total volume of 50 μL that contained 5 units Taq DNA polymerase (TakaRa, Dalian, China), 0.2 μM of each sense and antisense primer, 2.0 mM MgCl_2_, 200 μM dNTP, and 2 μL of template (2 ng/μL). Thermal cycling conditions involved an initial denaturation step at 95°C for 5 min, followed by 30 cycles of 95°C for 40 s, 40°C for 40 s, and 72°C for 3 min, and a final extension step at 72°C for 10 min. Polymerase chain reaction–amplified products were electrophoresed on a 1% agarose-gel, stained with ethidium bromide, and visualized under UV light to compare their lengths with DNA markers. Polymerase chain reaction products approximately covering their target regions were then sent out for Sanger sequencing (Sangon, Shanghai, China). The primers were listed as follows:

HAdV21-1-F: 5′-GTCATATCATAGTAGCCTGTCG-3′
HAdV21-1-R: 5′-GGAAGTTACGCTTGTTGG-3′
HAdV21-2-F: 5′-CCCTTGCTACCAAAGACC-3′
HAdV21-2-R: 5′-GCACTACAGCCATCATAAGC-3′
HAdV21-3-F: 5′-GGAGGCTCCCTTTGTACC-3′
HAdV21-3-R: 5′-ACCGCCACGGAAGCTATG-3′
HAdV21-4-F: 5′-GCATGGCTGGCAGTGGTA-3′
HAdV21-4-R: 5′-TGCATCTGGGCAACAAAA-3′
HAdV21-5-F: 5′-TTTCCCAGGCTTTCAGTT-3′
HAdV21-5-R: 5′-CAAGGCAGTCAATCAGTTCTA-3′
HAdV21-6-F: 5′-AATGGCATTGTATTTATGG-3′
HAdV21-6-R: 5′-TTTAGACTTTGCTGTGGC-3′
HAdV21-7-F: 5′-TTGGACACGGAAGTAGACAG-3′
HAdV21-7-R: 5′-CTAGCAGCATAGAATCAGTAAA-3′

### Bioinformatics Analysis

#### MinION Data Process

During MinION sequencing, Guppy embedded in MinKNOW (v3.3.2) performed the basecalling of raw signal event files to fastq reads. Reads with an average quality score ≥7 were labeled as “pass” files and were used for downstream analysis. Nanoplot (v1.22.0) (De Coster et al., 2018) was used to get an overview of the sequencing data including distributions of quality score and length. To calculate the percentages of reads belonging to the host, virus, bacteria, and archaea at the level of superkingdom, we employed Minimap2 (v2.16) (Li, 2018) and BLASTN (v2.8.1) to align the reads against reference genomes downloaded from National Center for Biotechnology Information (NCBI) RefSeq database as of March 21, 2019, including the human genome (Grch38.p12), virus genomes (*n* = 8,588), bacteria genomes (*n* = 12,668), and archaea genomes (*n* = 283). The parameters of Minimap2 in aligning to human and viral genomes were “-ax map-ont -k 15” and “-ax map-ont -k 7,” respectively. Samtools (v1.9) (Li, 2011) was used to handle the sam or bam files to extract aligned or unaligned reads for next-step alignment. Read depth across a reference genome was obtained by the “samtools depth” function.

Centrifuge (v1.0.4) (Kim et al., 2016) aligned the MinION reads to a database composed of human and viral reference genomes to obtain a preliminary summary of target viruses. Minimap2 and Samtools were then used to extract reads aligned to corresponding reference genomes. Canu (v1.8) (Koren et al., 2017) performed *de novo* assembly of selected reads to generate a draft genome sequence and Nanopolish (v0.11.0) (Loman et al., 2015) was then used to polish the draft sequence. Online BLASTN analysis was conducted to get the best hit of the draft genome sequence.

In order to investigate the minimal read number sufficient to identify present isolate, we downsampled the nanopore reads by randomly extracting reads at specific sequencing depths of 5, 10, 20, 30, 40, 50, 60, 70, 80, 90, 100, 200, 300, 400, 500, 1,000, 5,000, 10,000, 15,000, 20,000, 25,000, 30,000, and 35,000 for 1,000 times. The reads were aligned to the MinION genome sequence by Minimap2, and the alignment statistics were summarized by using Samtools and Perl scripts.

#### Illumina Data Process

Raw sequencing data were filtered with the following criteria: (1) reads having ≥40 bp with a quality score of less than 38 were removed; (2) reads having ≥10 N bases were removed; (3) reads having an overlap of ≥10 bp with adapters were removed; (4) reads aligning to human reference genome were removed. The clean data were then *de novo* assembled with SOAP denovo (v2.04) (Li et al., 2010) and SPAdes (Bankevich et al., 2012). CISA (Lin and Liao, 2013) was employed to integrate the contigs and scaffolds. The gaps in the scaffolds were filled with gapclose (v1.12).

#### *In silico* Restriction Enzyme Analysis

The virtual REA was performed with *Cis*SERS (Sharpe et al., 2016). Multiple genomes including the current genome, two independently reported HAdV21a genomes [HAdV21a LRTI-7 (GenBank accession no. KY307857.1) (Hage et al., 2014) and HAdV21a NHRC 10030 (KJ364586.1) (Kajon et al., 2015)], two independently reported HAdV21b genomes [HAdV21b OHT-006 (MF502426.1) (Philo et al., 2018), and HAdV21b NHRC 32389 (KJ364573.1) (Kajon et al., 2015)], and the HAdV21 prototype (KF528688.1) were selected as inputs. Four restriction enzymes *Bam*HI, *Kpn*I, *Xho*I, and *Pst*I were chosen to recognize the restriction sites of each genome with default parameters. Gel images containing restriction sites and molecular weights were generated by *Cis*SERS.

#### Phylogenetic Analyses

*Hexon* and *fiber* gene sequences of HAdV species B were first downloaded from NCBI, and repetitive sequences were then removed by USEARCH (V11.0.667).^1^ *E4* sequences were extracted from the corresponding reference genomes. Multiple alignment was conducted using the ClustalW method in MEGA X (v10.0.5) (Kumar et al., 2018). Phylogenetic trees were then constructed by using the neighbor-joining algorithm with a bootstrap value of 1,000.

#### Recombinant Event Detection

The multiple alignments of genome sequences of HAdV21p (KF528688.1), HAdV3 (AY599834.1), HAdV66 (JN860676.1), and current genome were performed using ClustalW method in MEGA X. The alignment file in fasta format was used as the input of bootscan analysis of SimPlot (V3.5.1)^2^ with the following parameters: window = 1,000 bp, step = 20 bp, GapStrip = on, reps = 500, distance model = Kimura, T/t = 2.0, and tree model = neighbor-joining. To detect a subtype-wide recombinant event related to *E4* gene, multiple alignment of gene sequences belonging to all available HAdV21a, HAdV21b, and HAdV21p, as well as HAdV3, was performed.

## Results

### A HAdV21a Strain Identified by MinION Sequencing

After a ∼27.5 h run, MinION generated 55,135 reads, among which 35,466 (64.33%) passed quality control (average base quality = 9.8, mean read length = 997.6 bp, total length = 35,382,056 bp). The first read was produced after 6 s upon the initialization of the sequencing. The top five longest reads all had a length >34.1 kb (34,927, 34,775, 34,209, 34,208, and 34,141 bp, respectively). Preliminary taxonomic analysis revealed that 60.30% (*n* = 21,387) and 19.32% (*n* = 6,852) of reads could be aligned to human and viral genomes separately, with 2.61% of reads aligning to bacterial genomes and the rest (17.77%) defined as unclassified sequences.

As the universal PCR primers for HAdV rather than other respiratory pathogens were tested positive, we mainly focused on viral detection from the nanopore reads. A rapid taxonomic analysis revealed that the top four taxonomic ranks with the highest number of unique reads against the NCBI refseq genomes were *Homo sapiens* (Taxonomy ID = 9606), human adenovirus 7 (Taxonomy ID = 10519), human mastadenovirus B (Taxonomy ID = 108098), and simian adenovirus 21 (Taxonomy ID = 198503). As the three identified AdVs all belong to the AdV species B, we speculated that the target virus should have high homology with members from this species.

A total of 5,887 reads could be aligned to the genomes of the above three AdVs and were then *de novo* assembled into a draft sequence containing 35,180 bp. BLASTN analysis revealed that this sequence had 99.53% identity against HAdV21 strain CDC V2148A (GenBank accession no. KJ364588.1, length = 35,371 bp) with a 100% query coverage. Moreover, among the reads longer than 10 kb (*n* = 637), 20 kb (*n* = 115), and 30 kb (*n* = 21), 94.82, 94.78, and 95.24% of reads could be aligned to this strain with a genome coverage ≥73.10, 75.93, and 82.27%, respectively. We then extracted 5,999 reads aligned to this specific strain and obtained a draft genome containing 35,364 bp, with the read depth across the draft genome ranging from 65 X to 737 X (Supplementary Figure S1). Online BLASTN analysis showed that the draft sequence had the highest identity of 99.88% (query coverage = 100%) against five HAdV21a strains (GenBank accession no. KY307857.1, KF802425.1, KY307859.1, KF577598.1, KF577597.1). The corresponding reference genome coverages were separately 99.98, 99.98, 99.98, 99.98, and 99.97%. As expected, PCR targeting the HAdV21 *hexon* gene was tested positive. We finally named this isolate as HAdV21a isolate human/CHN/BB/201903/21.

**FIGURE 1:**
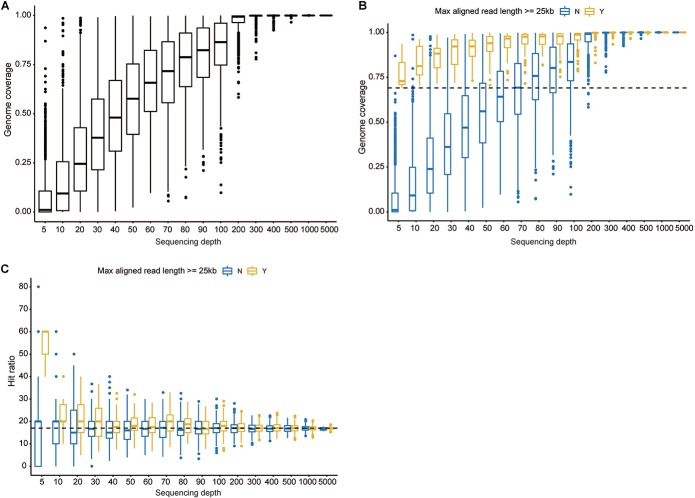
Investigation of the minimal read number to identify the current isolate. **(A)** The overall genome coverage distribution when 5–5,000 reads were randomly selected. The *x* axis denotes pseudosequencing depth, and the *y* axis, the corresponding genome coverages. **(B)** Genome coverage distribution decomposed by the maximal aligned read length. The dashed line corresponds to 69.10%. **(C)** Hit ratio distribution decomposed by the maximal aligned read length. The dashed line corresponds to 16.92%. “Y” represents read length being more than 25 kb, and “N” indicates less than 25 kb. Boxes represent the interquartile range (IQR) between the first and third quartiles (25th and 75th percentiles, respectively). Lines inside denote the median, and whiskers denote the most extreme values within 1.5 times IQR from the first and third quartiles. Outlier values are represented as points.

We further investigated the minimal read number sufficient to identify this isolate via a downsampling procedure. With respect to reference genome coverage (Figure 1A), a sequencing depth of 5 would generate an average genome coverage of 7.83% (standard deviation = 13.05%). When the depth gradually increased from 10 to 400, the minimal genome coverage ranged from 0 to 92.43%. A minimum of 500 reads could cover at least 96.49% of the reference genome as the sequencing depth increased. Notably, if more than 5,000 reads were randomly selected each time, the genome coverage was always 100% (Supplementary Figure S2). When decoding the genome coverage in the view of aligned read length, a downsampling procedure owning aligned reads with a maximal read length ≥25kb always had a genome coverage of ≥69.10% regardless of the sampling depth (Figure 1B). When sequencing depth was set to 500, aligned reads with a maximal read length ≥25 or <25 kb, respectively, obtained a ≥99.31% or ≥96.49% genome coverage. Moreover, 16.93%, on average, ranging from 16.69 to 17.32% for each sequencing depth, of reads could be aligned to the reference. Regardless of the maximal aligned read length, larger or smaller than 25 kb, the hit ratios (number of aligned reads divided by the corresponding sequencing depth) fluctuated around but finally converged to 16.93% (Figure 1C), which is close to the actual hit ratio of 16.92% (6,002/35,466).

### Genomic Discrepancies Between MinION and Illumina Sequencing

**FIGURE 2:**
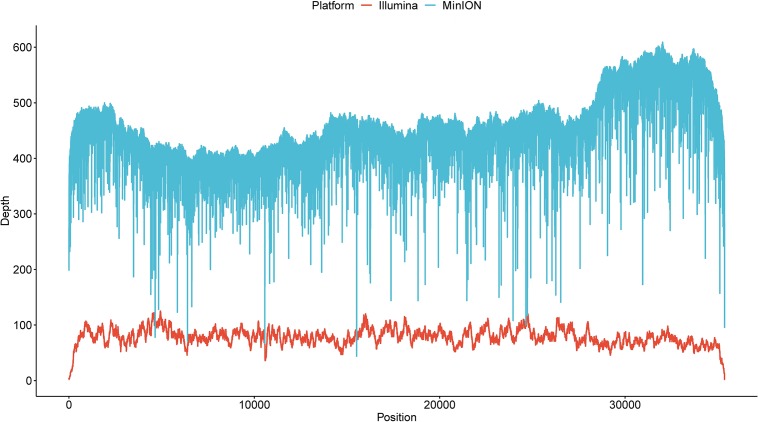
The read depth across the current genome by two sequencing platforms.

Illumina sequencing was further employed to *de novo* assemble the viral genome. After quality control, a total of 133,334 reads with a GC content of 44.04% were used for downstream assembly. One scaffold with a length of 35,369 bp was obtained. A total of 31 genomic discrepancies between MinION and Illumina sequencing were identified by both MEGA and dnadiff (Phillippy et al., 2008) programs (Table 1) and further validated and confirmed by PCR coupled with Sanger sequencing. Among these discrepancies, six ones were single-nucleotide polymorphisms (SNPs), and the rest belonged to insertion and deletions (indels). All the SNPs could be classified as base transitions. Notably, 19 of 25 indel loci in MinION data located next to homopolymeric regions composed of multiple adenines [poly(A)], thymines [poly(T)], guanines [poly(G)], or cytosines [poly(C)] (Supplementary Table S1). The dnadiff tool revealed that the pairwise nucleotide identity between the two draft sequences was 99.91%. Although sequencing errors still existed in the polished MinION genome, we are confident that the MinION output could achieve the goal of identifying pathogen at a comparable level. The corrected genome contained 35,369 bp with a GC content of 51.21%. The MinION read depth across the genome ranged from 44 X to 609 X, whereas the Illumina read depth ranged from 2 X to 125 X (Figure 2).

**TABLE 1:** Genomic discrepancies between MinION and Illumina sequencing.

**No.**	**Base in MinION assembly**	**Depth in MinION (X)**	**Base in Illumina assembly**	**Depth in Illumina (X)**	**Base in PCR product**
1	C	584	T	31	T
2	T	576	C	61	C
3	–	–	T	77	T
4	C	601	T	83	T
5	–	–	T	76	T
6	A	554	G	76	G
7	T	517	C	75	C
8	–	–	A	94	A
9	–	–	G	105	G
10	C	436	–	–	–
11	–	–	C	117	C
12	T	544	–	–	–
13	–	–	G	78	G
14	G	538	–	–	–
15	–	–	T	58	T
16	–	–	T	75	T
17	G	601	–	–	–
18	–	–	A	85	A
19	–	–	C	106	C
20	–	–	G	90	G
21	T	624	–	–	–
22	C	577	–	–	–
23	–	–	G	66	G
24	G	628	–	–	–
25	–	–	G	77	G
26	–	–	C	106	C
27	G	653	A	66	A
28	A	623	–	–	–
29	–	–	A	51	A
30	–	–	G	34	G
31	–	–	T	22	T

*“–” indicates a deletion in the sequence or not applicable in sequencing depth.*

### Genomic Characterization of HAdV21a Sequence

#### Genome Type Analysis

**FIGURE 3:**
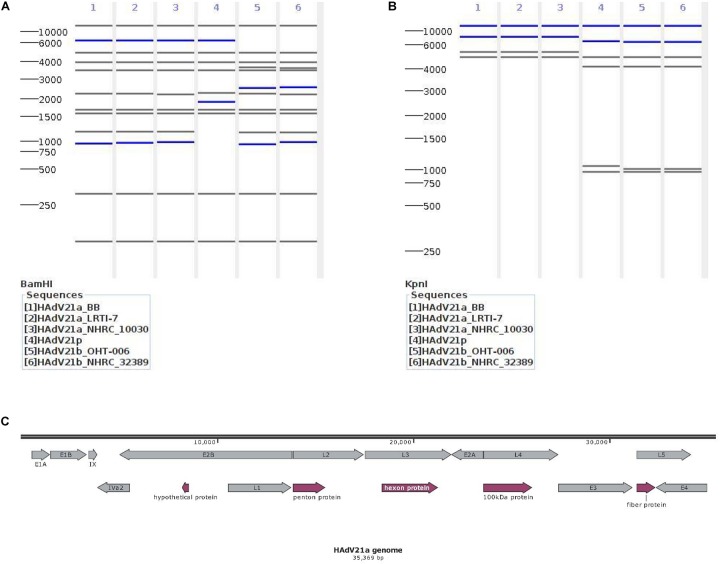
Genomic characterization of the current isolate. **(A,B)** Genome type analysis using restriction enzyme *Bam*HI **(A)** and *Kpn*I **(B)**. The numbers on the far left denote the molecular weight markers (bp). **(C)** Genomic annotation of the current isolate. Genes were displayed in gray, and CDSs discussed below in plum. “100 KDa protein” indicates the 100 kDa hexon-assembly associated protein.

In order to conduct a genome type analysis of our HAdV21a genome, a tool named as *Cis*SERS (Sharpe et al., 2016) that could perform *in silico* REA was used. REA using *Bam*HI (Figure 3A) and *Kpn*I (Figure 3B) identified our genome as a variant relative to the prototype and subtype 21b, the restriction band patterns of which were consistent with previous studies (Hage et al., 2014; Kajon et al., 2015). *Xho*I and *Pst*I merely discriminated HAdV21 prototype from subtype 21a and 21b (Supplementary Figure S3).

#### Genomic Annotation

A total of 13 genes were annotated from the genome sequence, all having ≥99.81% identities against the genes from reference genome KY307857.1 (Figure 3C). There were 48 coding sequences (CDSs) and 4 promoters located in these genes. The annotated hexon and fiber proteins that encode major neutralizing epitopes (Kajon et al., 2015) all had 100% identities with those of the reference KY307857.1.

#### Polymorphism Analysis

Multiple alignment coupled with polymorphism analysis was further conducted. A G → T conversion occurred in the hypothetical protein (GenBank accession no. APT35313.1) encoded by the *E2B* gene, leading to a conversion of proline to threonine in the deduced amino acid sequence. When comparing the promoter region from position 10,562 to 10,597 in the current genome with other HAdV21s, we found two poly(T) regions that were highly varying in the number of T bases (the first poly(T) region: 13 Ts in current genome vs. 9–16 Ts in other references; the second poly(T) region: 4 Ts in current genome vs. 4–5 Ts in other references). A similar variation in poly(T) regions was also observed in the upstream region of CDS for a 52 kDa protein encoded in the *L1* gene. One insertion of 6 bp and a 45 bp deletion were detected in the penton protein, and one 3 bp deletion and one 15 bp insertion were found in 100 kDa hexon assembly associated protein, which are shared features in subtype 21a (Hage et al., 2014) and 21b relative to the prototypes. Notably, some deletions and insertions occur in the intergeneric regions of CDSs; that is, an insertion of 6–12 bp (all As) between penton protein and protein VII precursor was detected in the present genome relative to all other references. Moreover, a deletion of 9 bp between protein VI precursor and hexon protein was found in comparisons of the current genome with the prototypes. Multiple polymorphisms were also discovered in the non-annotated 5′ region of the genome, such as a 1 bp deletion at position 242 and a conversion of C to T at position 252, the effects of which on viral life need further investigation.

#### Phylogenetic Analysis

**FIGURE 4:**
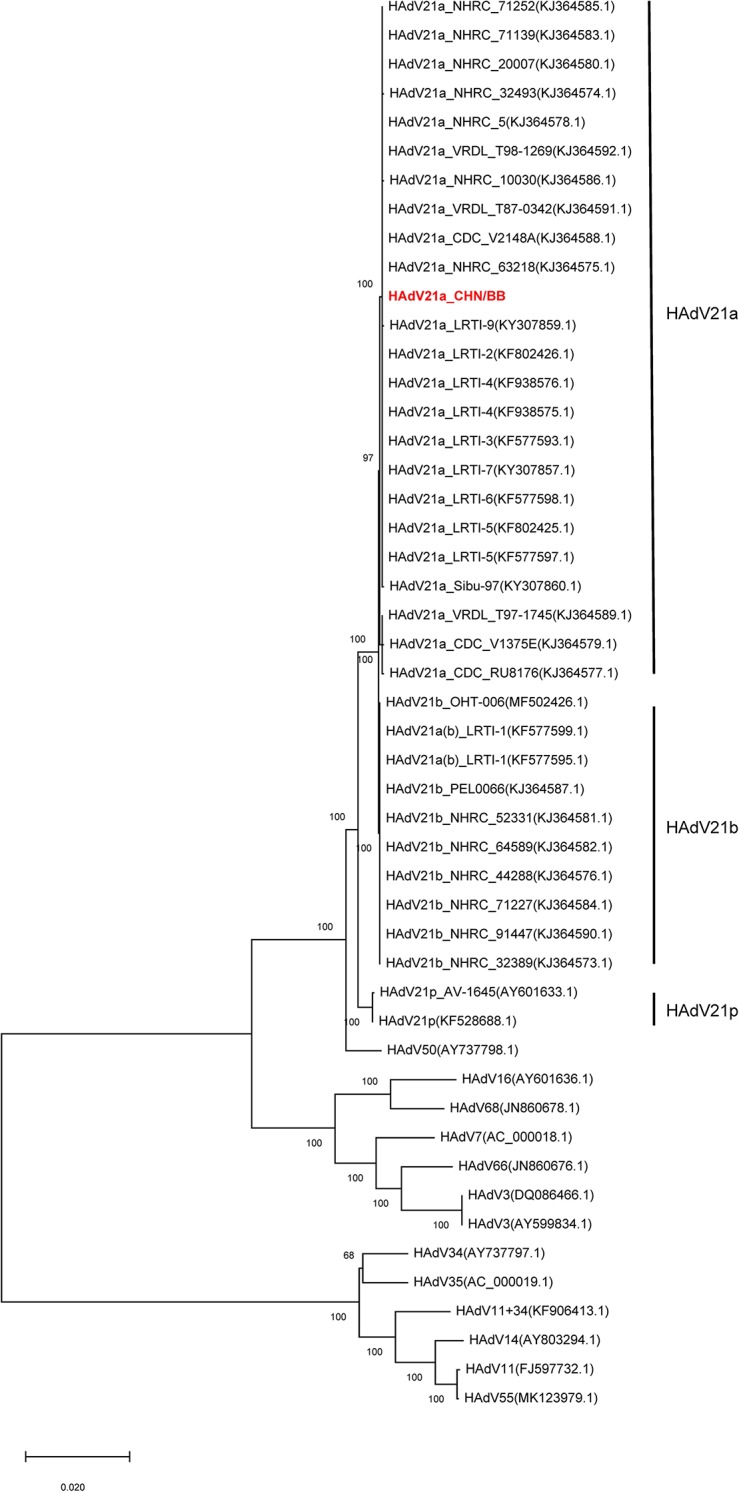
Phylogenetic tree constructed via whole-genome sequences. All available HAdV21 and other human mastadenovirus B genomes were used. The sequence displayed in red represents the current isolate.

Phylogenetic trees were constructed with *hexon*, *fiber*, and full genome sequences. *Hexon*- and *fiber*-based trees revealed that the current genome clustered with members from HAdV21a and 21b (Supplementary Figures S4, S5). In addition, the phylogenetic relationship between HAdV50 and HAdV21 was closer than with other subtypes. Phylogenetic trees via full-length genome sequences showed that the current sequence was placed within a clade composed of members from subtype 21a (Figure 4). The three subtypes, 21a, 21b, and 21p, were clearly separated. The prototype of HAdV50 was again observed to cluster with HAdV21.

### A Common Recombinant Event Shared by HAdV21a Strains

**FIGURE 5:**
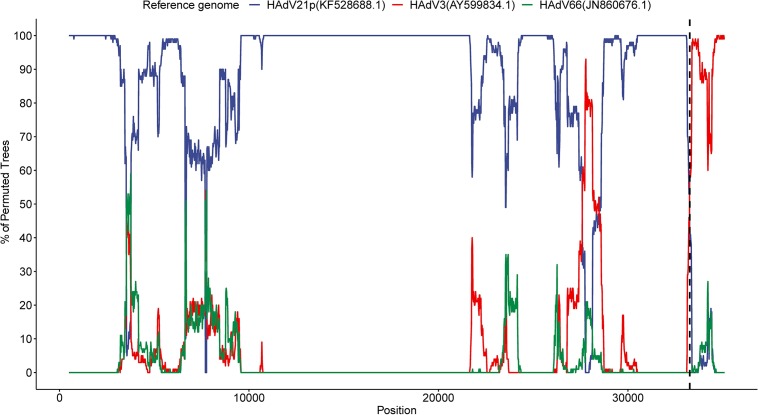
Bootscan analysis based on multiple alignment of the current isolate, HAdV3, HAdV21p, and HAdV66 genomes. The current genome was selected as the query sequence, whereas the rest were references. The dashed line represents the approximate position 33,260 in the present genome.

We detected a recombinant event approximately starting from position 33,260 in the present HAdV21a genome that indicated a recombinant *E4* gene originating from the HAdV3 prototype (Figure 5). BLASTN analysis revealed an identity of 99.54% between *E4* genes from the current isolate and the HAdV3 prototype, which decreased to a range of 93.85–98.88% when it was aligned to E4 region from other non-HAdV3 members. A phylogenetic tree constructed based on the *E4* gene sequences also revealed that all the strains in subtype 21a clustered with HAdV3 (Supplementary Figure S6). BLASTN identities of all available HAdV21a E4 genes against the HAdV3 *E4* gene ranged from 99.42 to 99.57%, which were higher than those of HAdV21p against HAdV3 (98.64%). Thus, the recombinant E4 region in HAdV21a strains should be a common feature that has never been reported before.

## Discussion

In this study, we report the first identification of HAdV21a in China via MinION and Illumina sequencers. This isolate contains 35,369 bp in the genome, encoding 13 genes and 48 CDSs. Genome type analysis *in silico* revealed that the present isolate could be distinguished from the prototype and subtype 21b. BLASTN and phylogenetic trees also confirmed this isolate belongs to subtype 21a. We further identified common features shared by HAdV21a members, such as insertions and deletions discovered in penton and 100 kDa hexon assembly associated proteins and a recombinant event in the *E4* gene, when compared with other HAdVs.

Nanopore sequencing technology has an advantage of extremely long-read output (>2.3 Mb according to ONT website) over current NGS platforms. This characteristic has promoted rapid identification of pathogens in the field (McIntyre et al., 2016; Castro-Wallace et al., 2017; Johnson et al., 2017; Edwards et al., 2019), especially for bacteria and viruses. Human adenoviruses usually have 35–36 kb in their genomes, which could be captured and covered by ONT with a single read. There were 21 MinION reads longer than 30 kb, 20 of which could be identified as HAdV21a with a genome coverage ≥82.27%. These reads were produced in 54 min to ∼15.5 h after the start of sequencing, with eight (40%) generated in ≤2.5 h. This demonstrated the feasibility of nanopore technology in the rapid detection of HAdV, thus providing key information for disease control and treatment.

Oxford Nanopore Technologies is hindered by its high sequencing error rate (10–15%). We observed 31 discrepancies between MinION and Illumina sequencing. Although indels and substitutions are frequent in nanopore data, there might be other factors involved in these discrepancies, that is, the read correction, read assembly, and genome polishing methods (Loman et al., 2015; Koren et al., 2017; Xiao et al., 2017), in addition to potential sequencing errors, which needs further investigation. Base modifications have been reported in human, prokaryotes, and viruses (Gokhale and Horner, 2017; Xiao et al., 2018), some of which could be detected by ONT (Simpson et al., 2017; Liu et al., 2019). Thus, base modifications may also contribute to genomic discrepancies (Greig et al., 2019). Illumina platforms usually have very low sequencing errors, however, they are characterized by a relatively complex sample preparation procedure, a relatively long sequencing time, and high-performance computing equipment for downstream analyses, which therefore limits the field application of these platforms under non-laboratory conditions. Considering that the pairwise nucleotide identity between MinION and Illumina sequences was 99.91% in our study, we believe that MinION correctly identified the pathogen despite sequencing errors. With the release of a new version of flow cell, ONT sequencing errors should dramatically decrease in the future.

Because of the long-read output by MinION, a minimum of 500 reads covered ≥96.49% of the present genome. This implied that under resource-limited conditions we could attempt to terminate the sequencing run ahead of time. It was observed that a downsampling procedure with maximal aligned read length ≥25 kb could cover ≥69.10% of the genome. As most reads longer than 20 kb could be identified as part of the current genome, we deemed reads with a length of 25 kb were able to reach a genome coverage of 70.82% (25/35.3) regardless of sequencing errors.

We observed varying numbers of T bases in poly(T) regions upstream of the 52 kDa protein CDS in the current isolate when comparing to other references. Deletions and insertions occurred in the intergeneric regions of CDSs and non-annotated 5′ region of the genome, the effects of which require further investigation. Polymorphisms in penton and 100 kDa hexon assembly–associated proteins were discovered in all 21a and 21b relative to the prototypes, which have partially been reported in a previous study (Hage et al., 2014). We did not observe polymorphisms in the remaining proteins, indicating that high conservation exists in most proteins of HAdV21a.

Another common feature shared by HAdV21a members is the recombinant *E4* gene originating from HAdV3. Products of the *E4* gene could regulate cellular functions and participate in viral DNA replication and RNA processing (Weitzman, 2005). Both phylogenetic and homology analyses provided credible evidence for this recombinant event. When this recombinant gene occurred in HAdV21a remains to be elucidated. It has been noted that highly virulent HAdV21a could cause severe pneumonia, acute respiratory disease, and even death (Hage et al., 2014). However, in our study, the patient had only slight symptoms of respiratory tract infection and recovered in 1 week, implying differential susceptibility of populations and individuals. The difference in virulence might lie in polymorphisms discovered merely in the present isolate.

In China, epidemic HAdV subtypes usually contain HAdV1, 3, 4, 7, 11, 14, and 55 rather than HAdV21. Our study indicates that HAdV21a infection cannot be neglected, although it is rarely reported. The pathogen has caused severe pneumonia and acute respiratory distress syndrome in several regions, thus indicating the need to avoid severe cases in China. Epidemiological profiles of HAdV21 subtypes in China should also be investigated with state-of-the-art technologies.

## Data Availability Statement

All sequence data have been submitted to Sequence Read Archive of NCBI with an accession number PRJNA587860. The data are also available upon request. The genome sequence of present isolate has been deposited in NCBI GenBank with accession number MN686206.

## Ethics Statement

The patient provided written informed consent upon enrollment. The study conformed to the ethical guidelines of the 1975 Declaration of Helsinki and was approved by the Institutional Review Board of the Center for Disease Control and Prevention of Eastern Theater Command.

## Author Contributions

CHW and JHZ conceived and designed the study. YH, HL, and QZ collected samples and patient information. YH, JJZ, and YL conducted the experiments. FY, YH, and CJW generated sequencing data. FY, PL, and TW analyzed and interpreted the data. FY and JJZ wrote the manuscript with contributions from all other authors. All authors reviewed and approved the final manuscript.

## Conflict of Interest

The authors declare that the research was conducted in the absence of any commercial or financial relationships that could be construed as a potential conflict of interest.

## Footnotes

1 https://www.drive5.com/usearch/download.html

2 https://sray.med.som.jhmi.edu/SCRoftware/simplot/

## Supplementary Material

FIGURE S1 Read depth across the MinION draft genome sequence.

FIGURE S2 Genome coverage corresponding to each downsampling procedure.

FIGURE S3 Genome type analysis using restriction enzyme *Xho*I **(A)** and *Pst*I **(B)**. The numbers on the far left denote the molecular weight marker (bp).

FIGURE S4 Phylogenetic tree constructed via *hexon* gene sequences. The sequence displayed in red represents current isolate.

FIGURE S5 Phylogenetic tree constructed via *fiber* gene sequences. The sequence displayed in red represents current isolate.

FIGURE S6 Phylogenetic tree constructed via *E4* gene sequences. The sequence displayed in red represents current isolate.

TABLE S1 Homopolymer sequences upstream of indel loci.
