# Supplementary figures and images for: First Identification of Human Adenovirus Subtype 21a in China With MinION and Illumina Sequencers
Source: Front Genet. 2020 Apr 7;11:285. doi: 10.3389/fgene.2020.00285 (PMC7155751; doi:10.3389/fgene.2020.00285)

Platform — MinION

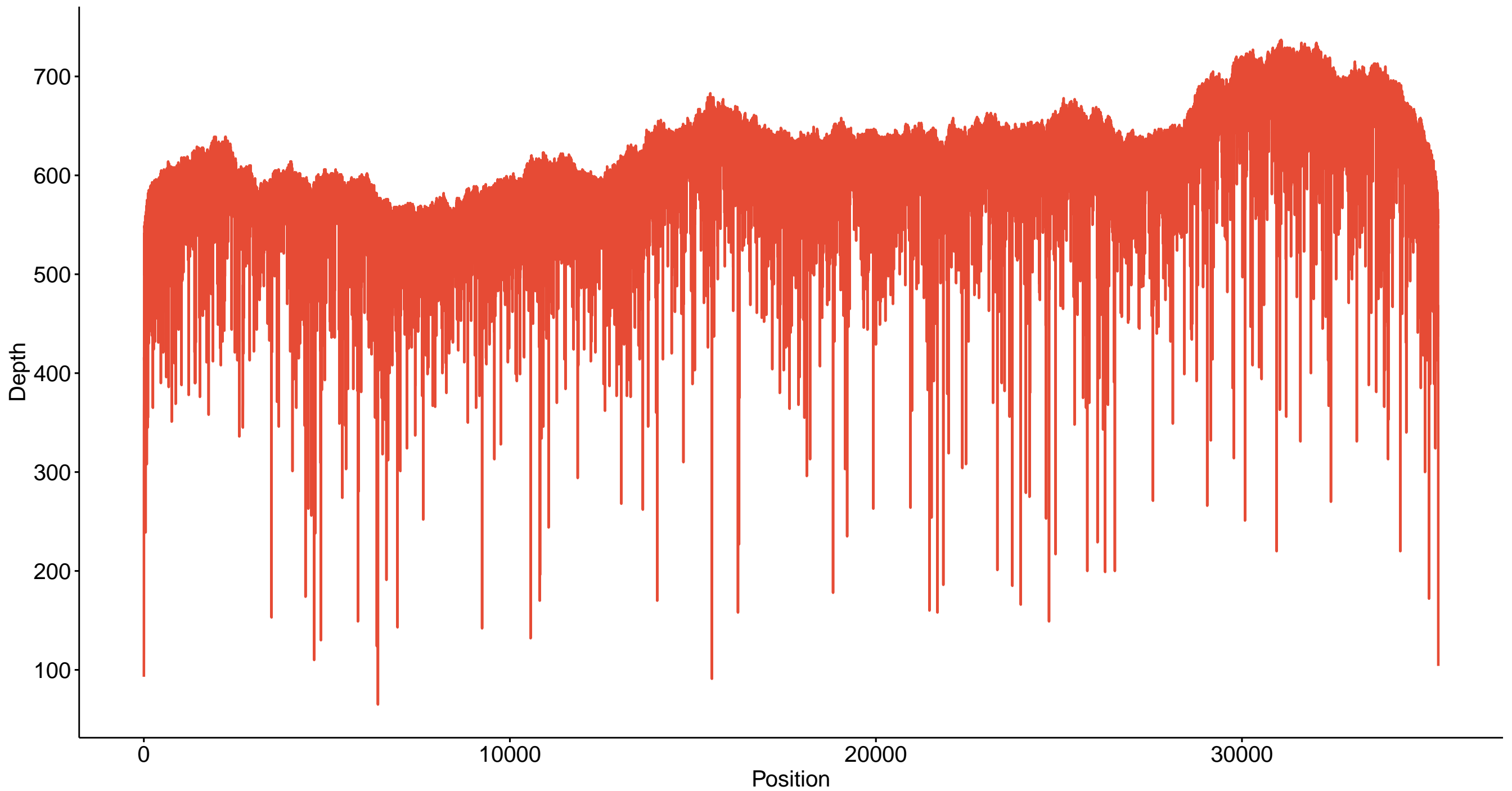

Supplement: FIGURE S1 — Read depth across the MinION draft genome sequence. [file Data_Sheet_1.PDF]

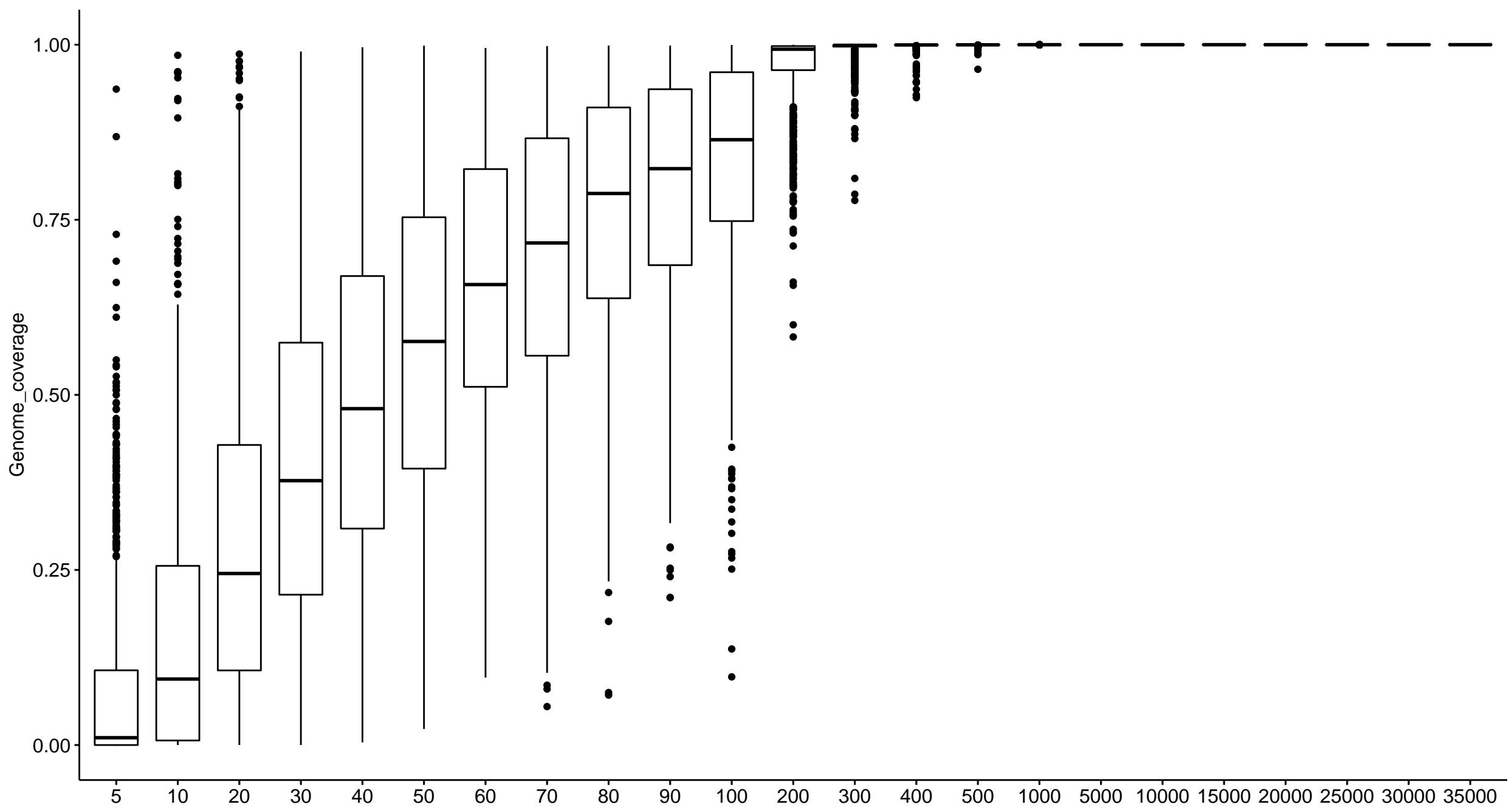

Supplement: FIGURE S2 — Genome coverage corresponding to each downsampling procedure. [file Data_Sheet_2.PDF]

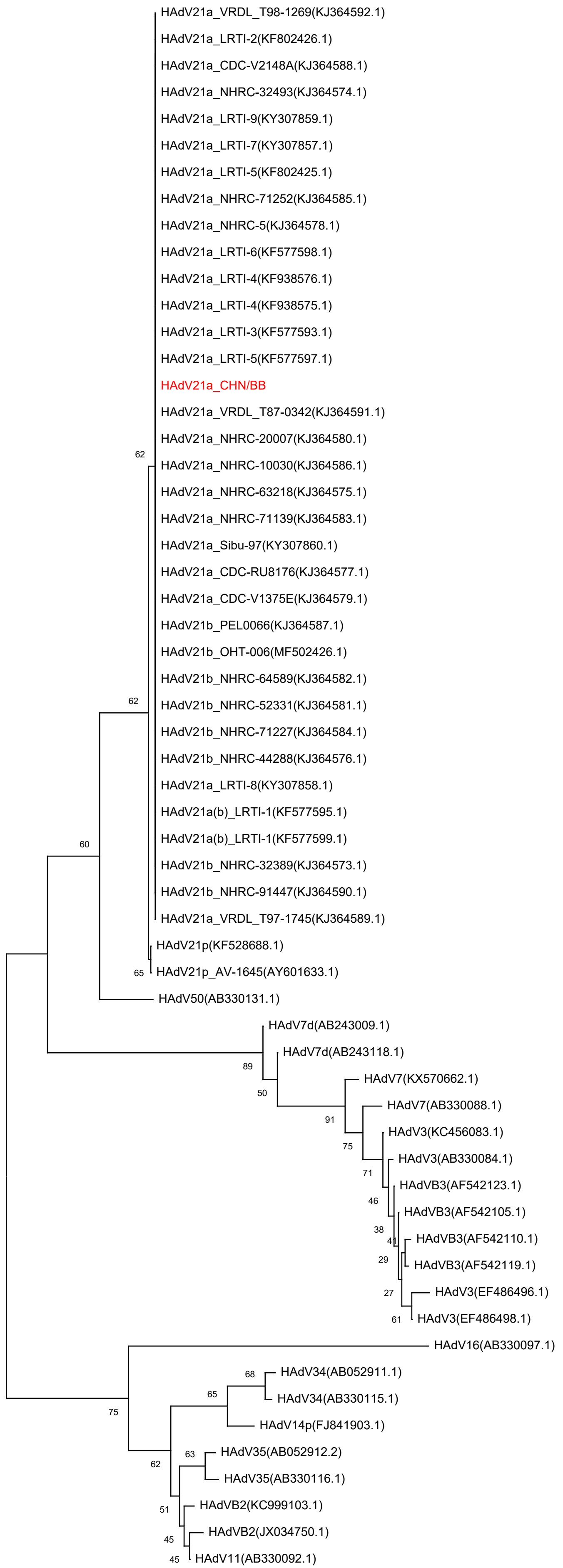

0.10

Supplement: FIGURE S4 — Phylogenetic tree constructed via hexon gene sequences. The sequence displayed in red represents current isolate. [file Data_Sheet_4.PDF]

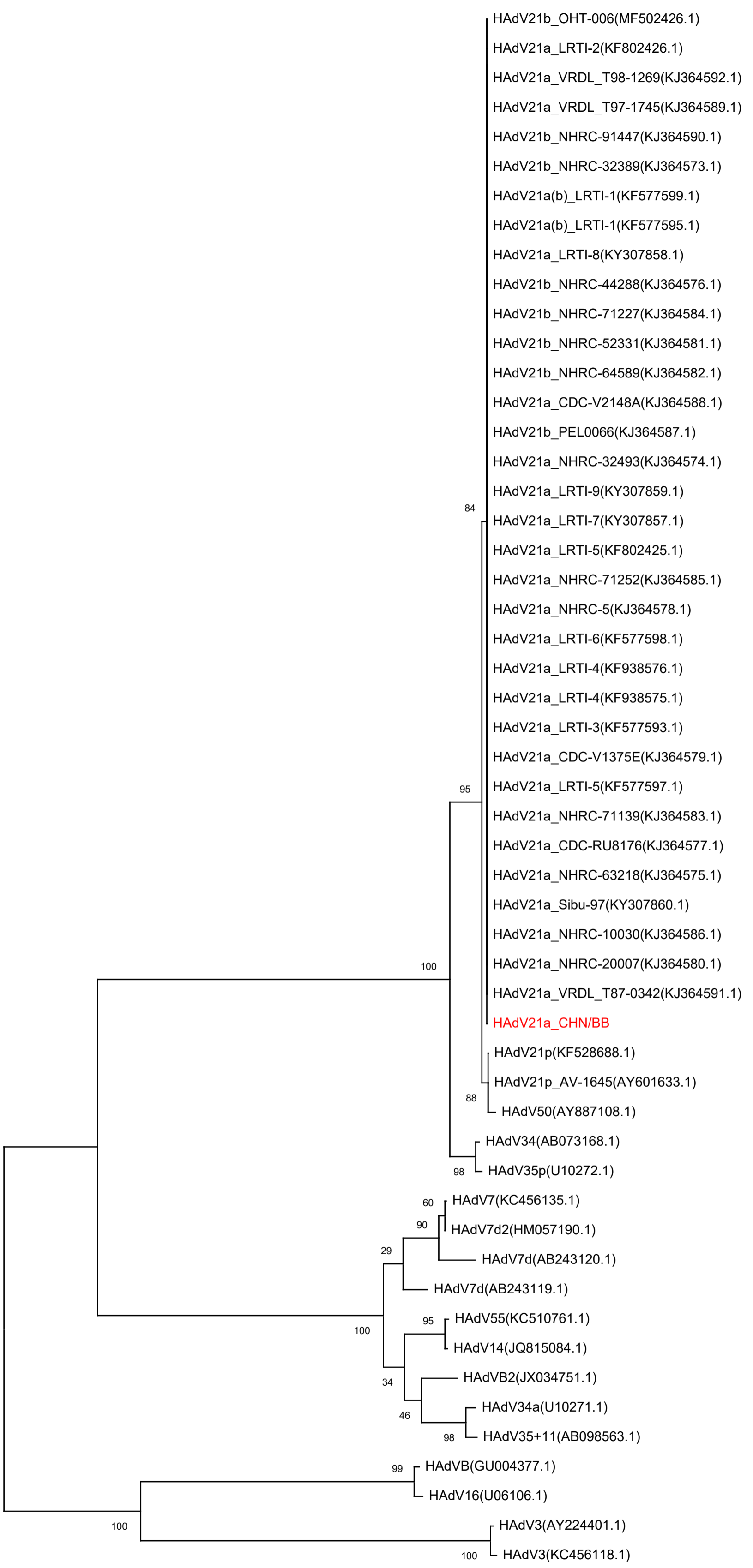

0.050

Supplement: FIGURE S5 — Phylogenetic tree constructed via fiber gene sequences. The sequence displayed in red represents current isolate. [file Data_Sheet_5.PDF]

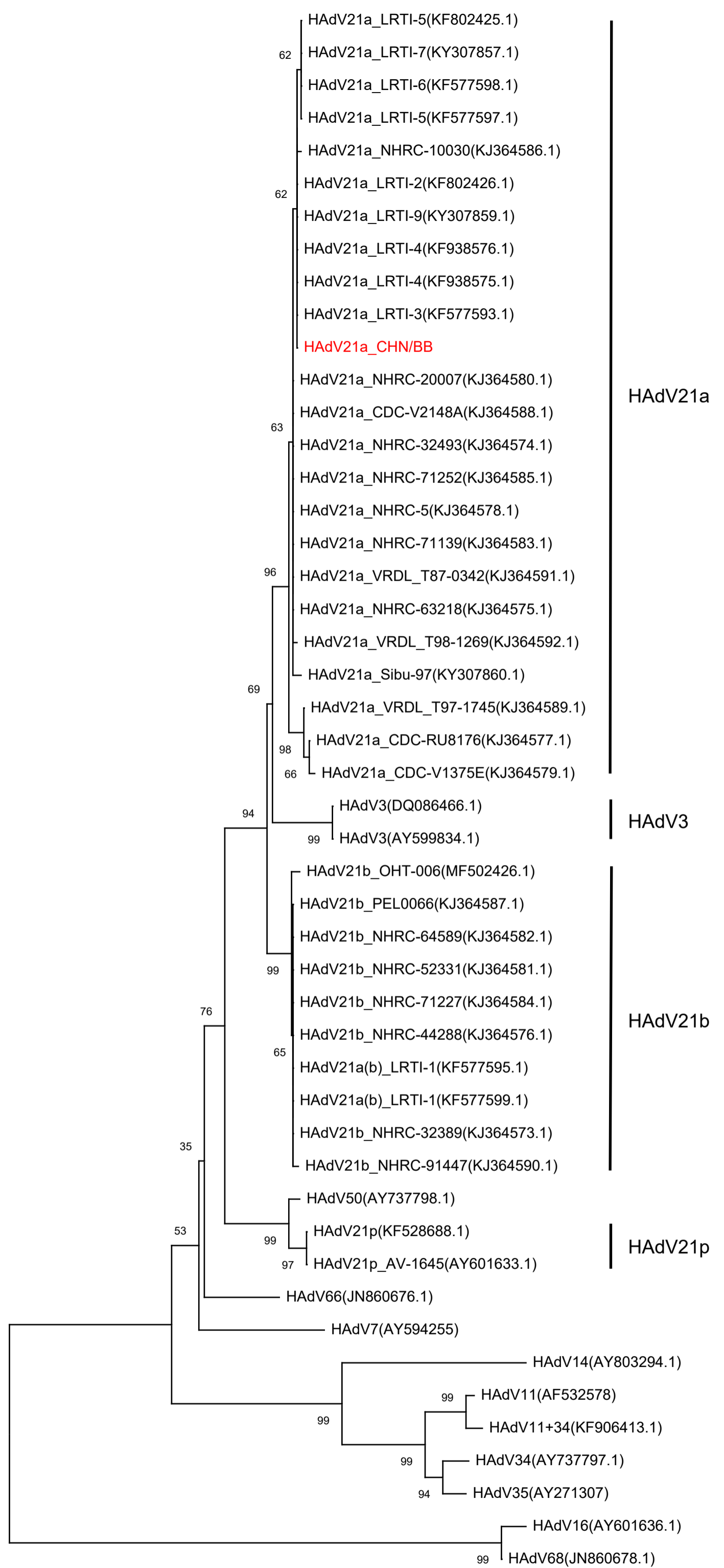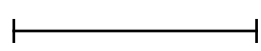

0.010

Supplement: FIGURE S6 — Phylogenetic tree constructed via E4 gene sequences. The sequence displayed in red represents current isolate. [file Data_Sheet_6.PDF]
