# Supplementary material for: First Identification of Human Adenovirus Subtype 21a in China With MinION and Illumina Sequencers
Source: Front Genet. 2020 Apr 7;11:285. doi: 10.3389/fgene.2020.00285 (PMC7155751; doi:10.3389/fgene.2020.00285)
Supplement: FIGURE S3 — Genome type analysis using restriction enzyme XhoI (A) and PstI (B). The numbers on the far left denote the molecular weight marker (bp). [file Data_Sheet_3.PDF]

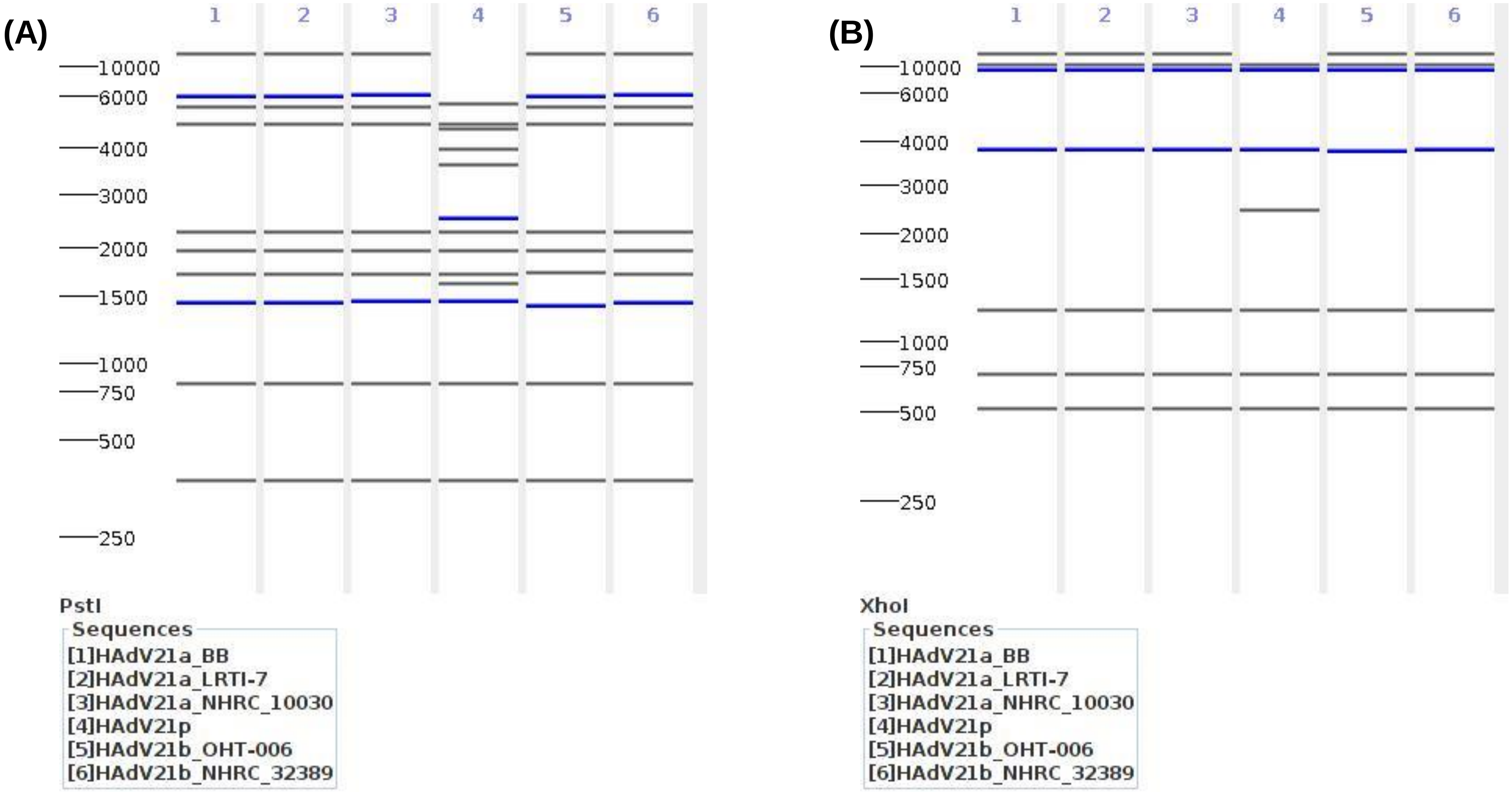
(A)
1
2
3
4
5
6
10000
6000
4000
3000
2000
1500
1000
750
500
250
PstI
Sequences
[1]HAdV21a_BB
[2]HAdV21a_LRTI-7
[3]HAdV21a_NHRC_10030
[4]HAdV21p
[5]HAdV21b_OHT-006
[6]HAdV21b_NHRC_32389
(B)
1
2
3
4
5
6
10000
6000
4000
3000
2000
1500
1000
750
500
250
XhoI
Sequences
[1]HAdV21a_BB
[2]HAdV21a_LRTI-7
[3]HAdV21a_NHRC_10030
[4]HAdV21p
[5]HAdV21b_OHT-006
[6]HAdV21b_NHRC_32389
