# Supplementary material for: First Identification of Human Adenovirus Subtype 21a in China With MinION and Illumina Sequencers
Source: Front Genet. 2020 Apr 7;11:285. doi: 10.3389/fgene.2020.00285 (PMC7155751; doi:10.3389/fgene.2020.00285)
Supplement: TABLE S1 — Homopolymer sequences upstream of indel loci. [file Data_Sheet_7.PDF]

Table S1. Homopolymer sequences upstream of indel loci.

No.	Base in MinION	Depth in MinION	Base in Illumina	Depth in Illumina	Base in PCR product	Homopolymer sequences upstream of indel loci
1	C	584	T	31	T	-
2	T	576	C	61	C	-
3	-	-	T	77	T	-
4	C	601	T	83	T	-
5	-	-	T	76	T	-
6	A	554	G	76	G	-
7	T	517	C	75	C	-
8	-	-	A	94	A	-
9	-	-	G	105	G	CCGAGGGGGG
10	C	436	-	-	-	TCCTCCCC
11	-	-	C	117	C	CTCTCCCC
12	T	544	-	-	-	TGGCTTTTT
13	-	-	G	78	G	-
14	G	538	-	-	-	AGGAGGGGG
15	-	-	T	58	T	GTCGTTTTTTTTTTT
16	-	-	T	75	T	CCTCTTTTTTT
17	G	601	-	-	-	CGGAGGGG
18	-	-	A	85	A	TTCTAAAAAAAAAAAAAAAAAA
19	-	-	C	106	C	CCCGCCCCC
20	-	-	G	90	G	GGCTGGGGG
21	T	624	-	-	-	GGACTTTTTT
22	C	577	-	-	-	-
23	-	-	G	66	G	CACCGGGGG
24	G	628	-	-	-	-
25	-	-	G	77	G	GCTTGGGGGG
26	-	-	C	106	C	AACTCCCCC
27	G	653	A	66	A	-
28	A	623	-	-	-	AGGCAAA
29	-	-	A	51	A	TAATGGG
30	-	-	G	34	G	CAGGAAAAA
31	-	-	T	22	T	GCACTTTTT

Notes: “-” indicates a deletion in the sequence or not applicable in sequencing depth or no homopolymer sequence detected.
